# Elevated C-reactive protein-to-albumin ratio is associated with early neurological deterioration in acute ischemic stroke: a retrospective cohort study

**DOI:** 10.3389/fneur.2026.1880425

**Published:** 2026-10-20

**Authors:** Zheng Zhou, Wenwen Xu, Zhaoping Wu, Tingting Duan, Ming Yang, Yueqi Xu, Junnan Ye

**Affiliations:** 1 Department of Hematology, The Quzhou Affiliated Hospital of Wenzhou Medical University, Quzhou People's Hospital, Quzhou, China; 2 Department of Neurosurgery, The Quzhou Affiliated Hospital of Wenzhou Medical University, Quzhou People's Hospital, Quzhou, China; 3 Department of Neurology, The Quzhou Affiliated Hospital of Wenzhou Medical University, Quzhou People's Hospital, Quzhou, China; 4 Department of Emergency, The Quzhou Affiliated Hospital of Wenzhou Medical University, Quzhou People's Hospital, Quzhou, China

**Keywords:** acute ischemic stroke, C-reactive protein-to-albumin ratio, early neurological deterioration, inflammatory biomarkers, non-linear modeling, risk stratification

## Abstract

**Background:**

Early neurological deterioration (END) is a frequent complication of acute ischemic stroke (AIS) that contributes to poor clinical outcomes. The C-reactive protein-to-albumin ratio (CAR) reflects systemic inflammation relative to albumin levels and may be associated with END. We evaluated the association between log_2_-transformed CAR and END in patients with AIS.

**Methods:**

We retrospectively analyzed 1,766 patients with AIS admitted between September 2016 to September 2022. END was defined as an increase of ≥2 points in the NIHSS motor item or total score within 72 h of admission. Associations between log₂-CAR and END were assessed using a multivariable logistic regression analysis. Generalized additive and two-piecewise regression models were used as exploratory analyses to assess potential non-linearity. ROC curves were used to compare CAR with established biomarkers and the A2DS2 score.

**Conclusion:**

Higher CAR was independently associated with END in this cohort. CAR’s strongest predictive advantage was observed when compared with the A2DS2 clinical score, whereas superiority over CRP alone was not demonstrated. The possible threshold at a raw CAR of approximately 0.020 is exploratory and requires independent validation.

## 1 Introduction

Acute ischemic stroke (AIS) remains a major cause of morbidity and mortality worldwide, accounting for approximately 80% of all stroke cases and imposing a substantial burden on healthcare systems and society (1, 2). Despite significant advances in acute management, including intravenous thrombolysis and endovascular thrombectomy, a considerable proportion of AIS patients experience early neurological deterioration (END) during hospitalization (3, 4). END, typically defined as an increase of ≥2 points in the NIHSS motor item score or total score within 72 h of stroke onset (5–7), occurs in 10–40% of cases and is strongly associated with an increased risk of poor functional outcomes, prolonged hospital stays, and higher mortality (7–9).

The pathophysiology of END is multifactorial, but mounting evidence highlights the pivotal role of systemic inflammation in mediating secondary brain injury and neurological decline after AIS (10, 11). The ischemic cascade triggers a robust inflammatory response, characterized by activation of innate immunity, blood–brain barrier disruption, and infiltration of peripheral immune cells into the brain parenchyma (12–14). This inflammatory milieu not only exacerbates neuronal damage and infarct expansion but also contributes to the development of complications such as cerebral edema, hemorrhagic transformation, and recurrent vascular events (15, 16). Accordingly, inflammatory biomarkers have been extensively investigated as potential predictors of stroke progression and outcomes.

Among these, C-reactive protein (CRP), a classical acute-phase reactant, has been widely studied and shown to correlate with stroke severity, infarct volume, and adverse outcomes (17, 18). However, the prognostic accuracy of CRP alone for predicting END remains limited, as it does not account for the patient’s nutritional or hepatic synthetic status, both of which are increasingly recognized as important determinants of stroke prognosis (19, 20). Albumin, a negative acute-phase protein, possesses antioxidant, anti-inflammatory, and anti-thrombotic properties, and hypoalbuminemia has been independently associated with an increased risk of poor outcomes and mortality in AIS, likely reflecting malnutrition, chronic disease burden, or impaired hepatic function (21).

The C-reactive protein-to-albumin ratio (CAR) is a composite marker that relates inflammatory activity, as reflected by CRP, to albumin concentration (22, 23). CAR captures inflammatory, nutritional, and hepatic factors simultaneously, but whether this translates into better discrimination than CRP alone must be tested empirically. CAR has been associated with outcomes in cardiovascular, infectious, and oncologic conditions (24–26). In stroke, higher CAR has been linked to mortality, hemorrhagic transformation, and poor functional outcomes (27–29). Its association with END and the shape of this association remain incompletely characterized.

The majority of previous studies of inflammatory markers and END have modeled linear associations (27, 30). Exploratory flexible models may reveal departures from linearity, but any candidate cut point requires formal model comparison and independent validation before it can be considered clinically significant.

We hypothesized that higher CAR would be independently associated with END in patients with AIS. We therefore aimed to: (1) evaluate the association between log₂-CAR and END; (2) explore, without presuming, possible departures from linearity; and (3) compare CAR’s discriminative performance with clinical and laboratory comparators.

## 2 Methods

### 2.1 Study design and participants

This single-center retrospective cohort study was conducted at the Quzhou Affiliated Hospital of Wenzhou Medical University and included consecutive patients with AIS admitted between September 2016 to September 2022. The protocol was approved by the Institutional Ethics Committee (approval no. 2023-151), which waived informed consent because of the retrospective design and use of de-identified data. This study was conducted in accordance with the Declaration of Helsinki. The institutional AIS registry used in this study overlaps in sampling frame and calendar period with cohorts used in previous studies of stroke-associated pneumonia (31, 32); however, the present study addressed a different outcome and used an END-specific analytical cohort.

The inclusion criteria were as follows: (1) a confirmed diagnosis of AIS within 24 h of symptom onset, with admission within the same time window; (2) brain magnetic resonance imaging (MRI) performed within 48 h of symptom onset, with radiological confirmation of acute infarction on diffusion-weighted imaging; (3) blood samples for CRP and albumin obtained within 48 h of symptom onset; and (4) complete clinical and laboratory data available for analysis.

During the study period, 2,150 patients with AIS were screened. At the initial data screening stage, 215 unique patients were excluded because of incomplete medical records (*n* = 112), missing laboratory data (*n* = 69), or loss to follow-up (*n* = 35), leaving 1,935 patients with complete data. The reason-specific counts at this stage were not mutually exclusive and therefore summed to 216, whereas 215 represented the number of unique patients excluded. An additional 169 patients were excluded because of hemorrhagic transformation (*n* = 45), pre-existing pneumonia at admission (*n* = 69), active malignancy (*n* = 35), or severe hepatic or renal dysfunction (*n* = 20), yielding a final analytical cohort of 1,766 patients. Missing baseline or follow-up National Institutes of Health Stroke Scale (NIHSS) assessments and death or discharge within 7 days were not treated as additional standalone exclusion categories. The same two-stage patient accounting is shown in Figure 1.

**Figure 1:**
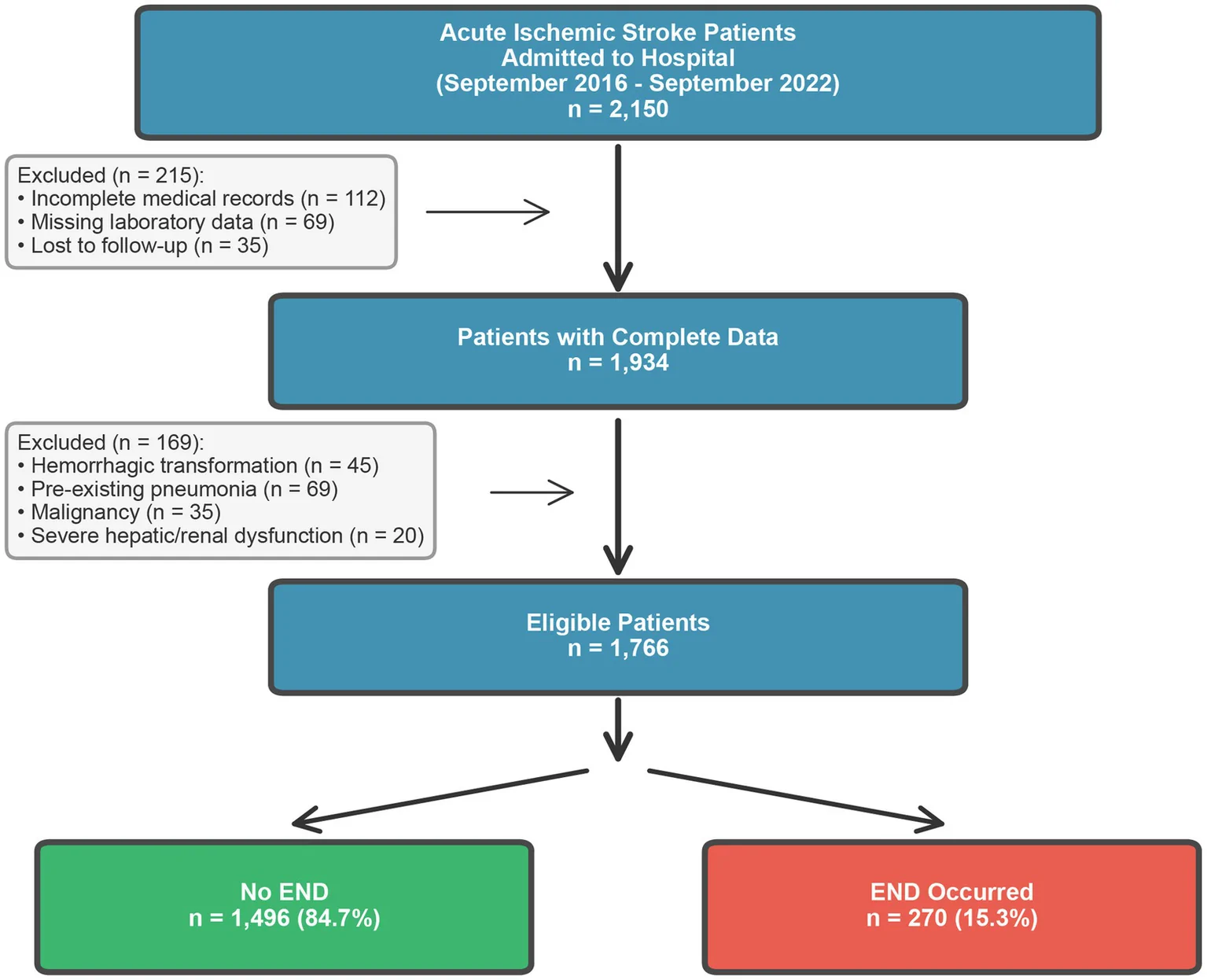
Patient selection flowchart. A total of 2,150 patients with AIS were screened. At the initial data screening stage, 215 unique patients were excluded because of incomplete medical records (*n* = 112), missing laboratory data (*n* = 69), or loss to follow-up (*n* = 35), leaving 1,935 patients with complete data. The first-stage reason counts were not mutually exclusive and summed to 216. An additional 169 patients were excluded because of hemorrhagic transformation (*n* = 45), pre-existing pneumonia at admission (*n* = 69), active malignancy (*n* = 35), or severe hepatic or renal dysfunction (*n* = 20), leaving 1,766 eligible patients. The final cohort comprised 1,496 patients without END and 270 with END. END, early neurological deterioration. END, early neurological deterioration.

### 2.2 Outcome definition

The primary outcome was END, defined as an increase of ≥2 points in the National Institutes of Health Stroke Scale (NIHSS) motor item score or total score within 72 h of admission compared with baseline, as assessed by certified neurologists blinded to laboratory data (5). Baseline NIHSS was assessed within 24 h of admission, and follow-up assessments were performed during the first 72 h.

### 2.3 Data collection and laboratory measurements

Baseline demographic and clinical data were extracted from electronic medical records, including age, sex, height, weight, body temperature, smoking status, and medical history (hypertension, diabetes mellitus, atrial fibrillation, and chronic obstructive pulmonary disease). At admission, stroke severity was assessed using the NIHSS (range, 0–42); dysphagia was evaluated using the Kubota Water Drinking Test (KWDT), with a score ≥3 indicating dysphagia (33); and consciousness was assessed using the Glasgow Coma Scale (range, 3–15) (34). Nasal feeding and disturbance of consciousness were also recorded.

Fasting blood samples were collected within 48 h of admission. Laboratory analyses included C-reactive protein (CRP), albumin, white blood cell count (WBC), neutrophil count (NTC), lymphocyte count (LTC), platelet count (PLT), fasting plasma glucose (FPG), glycated hemoglobin (HbA1c), lipid profile (total cholesterol, triglycerides, high-density lipoprotein cholesterol [HDL-C], and low-density lipoprotein cholesterol [LDL-C]), homocysteine (HCY), and renal function markers (uric acid, serum creatinine, and estimated glomerular filtration rate [eGFR] calculated using the CKD-EPI formula). All assays were performed in a central laboratory using standardized automated methods.

The C-reactive protein-to-albumin ratio (CAR) was calculated as CRP (mg/L) divided by albumin (g/L). To reduce skewness and facilitate interpretation, CAR values were log₂-transformed for all analyses.

### 2.4 Statistical analysis

Statistical analyses and data visualizations were conducted using Python (version 3.12.13; Python Software Foundation, https://www.python.org/). Continuous variables are presented as mean ± standard deviation or median (interquartile range), as appropriate, after assessment with the Shapiro–Wilk test. Categorical variables are presented as n (%). Between-group comparisons were performed using Student’s t-test or the Mann–Whitney U test for continuous variables, and the chi-squared or Fisher’s exact test was used for categorical variables, as appropriate.

Log₂-CAR was analyzed as a continuous variable and was categorized into quartiles. Multivariable logistic regression models were constructed to evaluate the association between log₂-CAR and END:

- Model 1: Unadjusted.
- Model 2: Adjusted for age and sex.
- Model 3: Further adjusted for smoking status, hypertension, diabetes mellitus, atrial fibrillation, COPD, baseline NIHSS score, dysphagia (KWDT score ≥3), baseline GCS score, white blood cell count, fasting plasma glucose, and estimated glomerular filtration rate.

Variance inflation factors (VIFs) were calculated to assess multicollinearity among covariates (VIF < 3 considered acceptable). Subgroup analyses examined the consistency of the CAR-END association across clinically relevant strata, with interaction terms also tested.

Generalized additive models with penalized splines and two-piecewise logistic regression were used to explore possible departures from linearity. A candidate cutoff was selected by maximizing model fit, and the two-piecewise model was compared with the linear model using a likelihood-ratio test. Because these analyses were exploratory, a non-significant model-comparison test was interpreted as insufficient evidence to establish non-linearity or a threshold effect.

Receiver operating characteristic (ROC) analysis was used to evaluate discrimination, and the areas under the curves (AUCs) were compared using DeLong’s test. The incremental value of CAR beyond A2DS2, CRP, WBC, and neutrophil count was assessed by comparing models with and without CAR. Two-sided *p*-values of <0.05 were considered statistically significant.

## 3 Results

### 3.1 Baseline characteristics

A total of 1,766 patients with AIS were included in the study after the two-stage exclusions, as shown in Figure 1; 270 (15.3%) patients developed END. Compared with patients without END, those with END were older (72.3 ± 12.0 vs. 68.7 ± 12.5 years; *p* < 0.001) and more often had atrial fibrillation (26.3% vs. 14.8%; *p* < 0.001). CAR (0.12 [0.05–0.44] vs. 0.07 [0.03–0.16]), CRP, and WBC were higher in the END group (all *p* < 0.001). Baseline NIHSS and Glasgow Coma Scale (GCS) score distributions also differed between the groups (both *p* < 0.001; Table 1); the median baseline NIHSS was 2.00 (1.00–4.00) in the END group and 3.00 (1.00–6.00) in the non-END group.

**Table 1:** Baseline characteristics of the study population stratified by END and non-END groups.

Variable	Overall (*N* = 1,766)	Non-END (*N* = 1,496)	END (*N* = 270)	*p*-value
Age (years)	69.26 ± 12.45	68.71 ± 12.45	72.30 ± 12.03	<0.001
Height (cm)	162.74 ± 8.79	162.85 ± 8.70	162.13 ± 9.30	0.237
Weight (kg)	68.73 ± 28.64	68.71 ± 29.20	68.79 ± 25.34	0.965
Temperature (°C)	36.50 (36.20–36.80)	36.50 (36.20–36.80)	36.40 (36.00–36.60)	<0.001
Gender, *n* (%)				0.059
Female	716 (40.54%)	592 (39.57%)	124 (45.93%)	
Male	1050 (59.46%)	904 (60.43%)	146 (54.07%)	
Smoke, *n* (%)				0.141
No	1117 (63.25%)	935 (62.50%)	182 (67.41%)	
Yes	649 (36.75%)	561 (37.50%)	88 (32.59%)	
Hypertension, *n* (%)				0.750
No	409 (23.16%)	349 (23.33%)	60 (22.22%)	
Yes	1357 (76.84%)	1147 (76.67%)	210 (77.78%)	
Diabetes, *n* (%)				0.190
No	1138 (64.44%)	974 (65.11%)	164 (60.74%)	
Yes	628 (35.56%)	522 (34.89%)	106 (39.26%)	
COPD, *n* (%)				0.409
No	1651 (93.49%)	1395 (93.25%)	256 (94.81%)	
Yes	115 (6.51%)	101 (6.75%)	14 (5.19%)	
Atrial fibrillation, *n* (%)				<0.001
No	1474 (83.47%)	1275 (85.23%)	199 (73.70%)	
Yes	292 (16.53%)	221 (14.77%)	71 (26.30%)	
Nasal feeding, *n* (%)				0.231
No	1589 (89.98%)	1352 (90.37%)	237 (87.78%)	
Yes	177 (10.02%)	144 (9.63%)	33 (12.22%)	
Disturbance of consciousness, *n* (%)				0.359
No	1642 (92.98%)	1395 (93.25%)	247 (91.48%)	
Yes	124 (7.02%)	101 (6.75%)	23 (8.52%)	
Dysphagia, *n* (%)				0.539
No	1541 (87.26%)	1309 (87.50%)	232 (85.93%)	
Yes	225 (12.74%)	187 (12.50%)	38 (14.07%)	
Laboratory parameters				
FPG (mg/dL)	101.81 (90.10–128.80)	100.91 (89.74–127.18)	106.23 (93.12–140.69)	0.003
HbA1C (%)	6.00 (5.50–7.20)	6.00 (5.50–7.20)	6.05 (5.50–7.20)	0.579
TG (mg/dL)	112.93 (79.93–155.00)	113.81 (81.48–156.99)	104.51 (73.51–146.14)	0.048
TC (mmol/L)	4.38 ± 1.10	4.36 ± 1.08	4.50 ± 1.17	0.084
HDLC (mmol/L)	1.17 ± 0.32	1.17 ± 0.31	1.19 ± 0.34	0.371
LDLC (mmol/L)	2.85 ± 1.00	2.83 ± 0.98	3.00 ± 1.10	0.014
UA (umol/L)	311.95 (251.00–380.00)	310.85 (252.25–376.60)	317.80 (250.65–397.85)	0.195
HCY (umol/L)	14.93 (11.71–19.20)	14.60 (11.41–19.00)	17.00 (13.65–20.00)	<0.001
CRP (mg/L)	2.82 (1.15–6.87)	2.50 (1.07–6.00)	4.33 (2.00–15.21)	<0.001
Albumin (g/L)	37.95 ± 3.85	38.08 ± 3.59	37.25 ± 5.00	0.009
CAR	0.07 (0.03–0.18)	0.07 (0.03–0.16)	0.12 (0.05–0.44)	<0.001
WBC (*10⁹/L)	6.97 (5.60–8.68)	6.89 (5.51–8.48)	7.81 (6.16–9.96)	<0.001
NTC (*10⁹/L)	4.54 (3.49–6.18)	4.42 (3.41–5.90)	5.30 (4.01–7.40)	<0.001
LTC (*10⁹/L)	1.54 (1.02–2.21)	1.52 (1.00–2.17)	1.82 (1.14–2.54)	<0.001
PLT (*10⁹/L)	189.00 (151.00–233.00)	189.00 (151.00–232.25)	183.50 (151.00–233.00)	0.868
HBG (g/L)	133.96 ± 18.11	134.51 ± 17.57	130.92 ± 20.60	0.007
BUN (mmol/L)	5.20 (4.17–6.46)	5.14 (4.12–6.36)	5.52 (4.32–6.70)	0.006
SCR (umol/L)	70.00 (58.52–84.00)	70.00 (59.00–83.93)	69.10 (56.62–86.83)	0.812
EGFR (ml/min/1.73 m²)	100.78 ± 34.32	100.84 ± 33.07	100.49 ± 40.66	0.895
Clinical characteristics				
SBP (mmHg)	151.00 (138.00–167.75)	150.00 (137.00–167.00)	153.00 (139.25–168.00)	0.078
DBP (mmHg)	82.00 (73.00–90.00)	82.00 (73.75–90.00)	82.00 (73.00–91.00)	0.989
NIHSS	3.00 (1.00–6.00)	3.00 (1.00–6.00)	2.00 (1.00–4.00)	<0.001
GCS	15.00 (14.00–15.00)	15.00 (14.00–15.00)	15.00 (13.00–15.00)	<0.001
A2DS2	3.00 (2.00–4.00)	2.00 (2.00–4.00)	3.00 (2.00–4.00)	0.269
KWDT	1.00 (1.00–1.00)	1.00 (1.00–1.00)	1.00 (1.00–1.00)	0.944

Data are presented as mean ± SD, median (interquartile range), or *n* (%). *p*-values were calculated using Student’s *t*-test, Mann–Whitney U test, or chi-square test as appropriate. END, early neurological deterioration (defined as an increase of ≥2 points in NIHSS motor item score or total score within 72 hours of admission); COPD, chronic obstructive pulmonary disease; FPG, fasting plasma glucose; HbA1C, hemoglobin A1c; TG, triglyceride; TC, total cholesterol; HDLC, high-density lipoprotein cholesterol; LDLC, low-density lipoprotein cholesterol; UA, uric acid; HCY, homocysteine; CRP, C-reactive protein; CAR, C-reactive protein to albumin ratio; WBC, white blood cell; NTC, neutrophil count; LTC, lymphocyte count; PLT, platelet; BUN, blood urea nitrogen; SCR, serum creatinine; EGFR, estimated glomerular filtration rate; NIHSS, National Institutes of Health Stroke Scale; GCS, Glasgow Coma Scale; KWDT, Kubota Water Drinking Test; SBP, systolic blood pressure; DBP, diastolic blood pressure; HBG, hemoglobin; A2DS2, Age, Atrial fibrillation, Dysphagia, Sex, Stroke Scale.

### 3.2 Baseline characteristics by log₂-CAR quartiles

Patient characteristics across log₂-CAR quartiles are shown in Table 2. The incidence of END was 50/508 (9.84%) in Q1, 44/396 (11.11%) in Q2, 74/431 (17.17%) in Q3, and 102/431 (23.67%) in Q4 (*p* < 0.001 for trend). These counts and percentages were recalculated from the denominators in Table 2 and were internally consistent. Higher CAR quartiles were associated with older age, more atrial fibrillation, higher inflammatory markers, and differences in clinical severity measures (all *p* < 0.001). Albumin decreased while CRP increased across quartiles, consistent with the CAR definition.

**Table 2:** Baseline characteristics of patients stratified by log₂-CAR quartiles.

Variable	Q1 (*N* = 508)	Q2 (*N* = 396)	Q3 (*N* = 431)	Q4 (*N* = 431)	*p*-value
END	50 (9.84%)	44 (11.11%)	74 (17.17%)	102 (23.67%)	<0.001
Age (years)	66.53 ± 11.84	68.30 ± 12.22	69.80 ± 12.41	72.81 ± 12.52	<0.001
Temperature (°C)	36.50 (36.20–36.70)	36.50 (36.20–36.70)	36.50 (36.20–36.75)	36.50 (36.20–36.80)	0.003
Gender, *n* (%)					0.296
Female	202 (39.76%)	151 (38.13%)	191 (44.32%)	172 (39.91%)	
Male	306 (60.24%)	245 (61.87%)	240 (55.68%)	259 (60.09%)	
Smoke, *n* (%)					0.012
No	324 (63.78%)	230 (58.08%)	297 (68.91%)	266 (61.72%)	
Yes	184 (36.22%)	166 (41.92%)	134 (31.09%)	165 (38.28%)	
Hypertension, *n* (%)					0.092
No	124 (24.41%)	73 (18.43%)	105 (24.36%)	107 (24.83%)	
Yes	384 (75.59%)	323 (81.57%)	326 (75.64%)	324 (75.17%)	
Diabetes, *n* (%)					0.560
No	325 (63.98%)	267 (67.42%)	274 (63.57%)	272 (63.11%)	
Yes	183 (36.02%)	129 (32.58%)	157 (36.43%)	159 (36.89%)	
Atrial fibrillation, *n* (%)					<0.001
No	470 (92.52%)	347 (87.63%)	345 (80.05%)	312 (72.39%)	
Yes	38 (7.48%)	49 (12.37%)	86 (19.95%)	119 (27.61%)	
Nasal feeding, *n* (%)					<0.001
No	501 (98.62%)	383 (96.72%)	399 (92.58%)	306 (71.00%)	
Yes	7 (1.38%)	13 (3.28%)	32 (7.42%)	125 (29.00%)	
Disturbance of consciousness, *n* (%)					<0.001
No	505 (99.41%)	388 (97.98%)	409 (94.90%)	340 (78.89%)	
Yes	3 (0.59%)	8 (2.02%)	22 (5.10%)	91 (21.11%)	
COPD, *n* (%)					<0.001
No	484 (95.28%)	377 (95.20%)	416 (96.52%)	374 (86.77%)	
Yes	24 (4.72%)	19 (4.80%)	15 (3.48%)	57 (13.23%)	
Dysphagia, *n* (%)					<0.001
No	483 (95.08%)	376 (94.95%)	390 (90.49%)	292 (67.75%)	
Yes	25 (4.92%)	20 (5.05%)	41 (9.51%)	139 (32.25%)	
Laboratory parameters					
FPG (mg/dL)	98.84 (88.30–120.69)	99.74 (90.10–122.54)	101.45 (90.10–131.10)	108.66 (91.90–139.84)	<0.001
HbA1C (%)	6.09 (5.50–7.13)	6.00 (5.50–7.10)	6.00 (5.50–7.10)	6.00 (5.52–7.45)	0.813
TG (mg/dL)	117.80 (82.37–164.07)	118.68 (86.58–162.52)	114.26 (82.37–161.64)	95.66 (69.08–132.85)	<0.001
TC (mmol/L)	4.30 ± 1.06	4.49 ± 0.98	4.52 ± 1.16	4.25 ± 1.15	<0.001
LDLC (mmol/L)	2.76 ± 0.96	2.94 ± 0.91	2.97 ± 1.03	2.77 ± 1.07	<0.001
UA (umol/L)	303.30 (250.00–379.25)	315.00 (257.73–386.55)	319.20 (257.10–383.10)	310.90 (244.50–372.75)	0.385
HCY (umol/L)	14.45 (10.90–18.86)	14.20 (11.70–18.10)	15.50 (11.95–19.65)	16.03 (12.50–20.05)	<0.001
CRP (mg/L)	0.73 (0.45–1.00)	2.00 (1.71–2.30)	4.00 (3.41–5.21)	17.50 (10.93–38.31)	<0.001
Albumin (g/L)	39.10 ± 3.70	38.55 ± 3.27	37.78 ± 3.53	36.23 ± 4.19	<0.001
WBC (*10⁹/L)	6.21 (5.10–7.52)	6.69 (5.52–7.94)	7.10 (5.70–8.73)	8.78 (6.93–11.20)	<0.001
NTC (*10⁹/L)	3.85 (3.08–4.87)	4.21 (3.33–5.10)	4.68 (3.60–6.09)	6.50 (4.69–8.96)	<0.001
LTC (*10⁹/L)	1.44 (0.93–2.04)	1.53 (1.04–2.12)	1.56 (1.07–2.26)	1.66 (1.04–2.54)	<0.001
PLT (*10⁹/L)	186.00 (149.00–228.00)	187.00 (152.00–228.25)	191.00 (153.00–231.50)	192.00 (152.00–242.00)	0.172
EGFR (ml/min/1.73 m²)	104.11 ± 32.40	102.51 ± 31.52	101.00 ± 35.56	95.06 ± 37.05	<0.001
Clinical characteristics					
NIHSS	2.00 (1.00–4.00)	2.00 (1.00–5.00)	2.00 (1.00–5.00)	4.00 (2.00–11.00)	<0.001
GCS	15.00 (15.00–15.00)	15.00 (15.00–15.00)	15.00 (14.00–15.00)	14.00 (12.00–15.00)	<0.001
A2DS2	2.00 (2.00–3.00)	2.00 (2.00–4.00)	3.00 (2.00–4.00)	4.00 (2.00–6.00)	<0.001
KWDT	1.00 (1.00–1.00)	1.00 (1.00–1.00)	1.00 (1.00–1.00)	1.00 (1.00–4.00)	<0.001

Data are presented as mean ± standard deviation (SD), median (interquartile range [IQR]), or number (percentage). *p*-values were calculated using one-way ANOVA or Kruskal-Wallis test for continuous variables, and chi-square test for categorical variables. Q1–Q4 represent quartiles 1–4 of log₂-transformed CAR (Q1: lowest, Q4: highest). *p* for trend was calculated for END across quartiles. CAR, C-reactive protein to albumin ratio; END, early neurological deterioration (defined as an increase of ≥2 points in NIHSS motor item score or total score within 72 hours of admission); Q, quartile; COPD, chronic obstructive pulmonary disease; FPG, fasting plasma glucose; HbA1C, glycated hemoglobin A1c; TG, triglycerides; TC, total cholesterol; LDLC, low-density lipoprotein cholesterol; UA, uric acid; HCY, homocysteine; CRP, C-reactive protein; Albumin, albumin; WBC, white blood cell count; NTC, neutrophil count; LTC, lymphocyte count; PLT, platelet count; EGFR, estimated glomerular filtration rate; NIHSS, National Institutes of Health Stroke Scale; GCS, Glasgow Coma Scale; A2DS2, Age-Atrial fibrillation-Dysphagia-Sex-Stroke severity score; KWDT, Kubota Water Drinking Test.

### 3.3 Association between log₂-CAR and END

Multivariable logistic regression analysis results are presented in Table 3. In the unadjusted model, patients in Q4 had a significantly higher risk of END than those in Q1 (OR = 2.90, 95% CI: 2.01–4.01, *p* = 0.0010). This association persisted after adjustment for age and sex (Model 2: OR = 2.66, 95% CI: 1.78–3.86, *p* = 0.0010). In the fully adjusted model (Model 3), the association remained statistically significant, although attenuated (Q4 vs. Q1: OR = 1.95, 95% CI: 1.27–2.88, *p* = 0.0010). A significant dose–response relationship was observed across quartiles (*p* for trend = 0.0010).

**Table 3:** Multivariable logistic regression analysis of the association between log₂-transformed CAR and early neurological deterioration.

Model	Exposure	OR (95% CI)	*p*-value	*p*-trend
Model 1	Log₂-CAR (continuous)	1.21 (1.13, 1.29)	0.0010	0.0010
	Q1	1.0 (Reference)		
	Q2	1.18 (0.80, 1.68)	0.5000	
	Q3	1.96 (1.35, 2.80)	0.0010	
	Q4	2.90 (2.01, 4.01)	0.0010	
Model 2	Log₂-CAR (continuous)	1.19 (1.11, 1.27)	0.0010	0.0010
	Q1	1.0 (Reference)		
	Q2	1.15 (0.71, 1.68)	0.5000	
	Q3	1.84 (1.21, 2.71)	0.0010	
	Q4	2.66 (1.78, 3.86)	0.0010	
Model 3	Log₂-CAR (continuous)	1.12 (1.03, 1.21)	0.0010	0.0010
	Q1	1.0 (Reference)		
	Q2	1.02 (0.62, 1.52)	0.5000	
	Q3	1.56 (1.01, 2.30)	0.0010	
	Q4	1.95 (1.27, 2.88)	0.0010	

Model 1: Unadjusted. Model 2: Adjusted for age and sex. Model 3: Fully adjusted for age, sex, smoking status, hypertension, diabetes mellitus, atrial fibrillation, chronic obstructive pulmonary disease, dysphagia, National Institutes of Health Stroke Scale score, Glasgow Coma Scale score, white blood cell count, fasting plasma glucose, and estimated glomerular filtration rate. The log₂-transformed CAR values were analyzed both as a continuous variable and categorized into quartiles (Q1–Q4). Q1 represents the lowest quartile and serves as the reference group. P-trend values were calculated by treating quartile categories as continuous variables in the logistic regression models. Odds ratios (ORs) and 95% confidence intervals (CIs) were estimated using the bootstrap method with 500 iterations. CAR, C-reactive protein to albumin ratio; END, early neurological deterioration; OR, odds ratio; CI, confidence interval; Q, quartile.

When analyzed as a continuous variable, each 1-unit increase in log₂-CAR was associated with an increased risk of END in all models: unadjusted OR = 1.21 (95% CI: 1.13–1.29), age- and sex-adjusted OR = 1.19 (95% CI: 1.11–1.27), and fully adjusted OR = 1.12 (95% CI: 1.03–1.21) (all *p* = 0.0010).

### 3.4 Subgroup analyses

Subgroup analyses showed generally positive point estimates for the association between log₂-CAR and END, although several stratum-specific confidence intervals included the null (Figure 2). No interaction test was statistically significant (all *p* for interaction >0.05), providing no evidence that the association differed across the examined strata. The subgroup results should therefore be interpreted as consistency analyses rather than evidence of a statistically significant association within every subgroup (Table 4).

**Figure 2:**
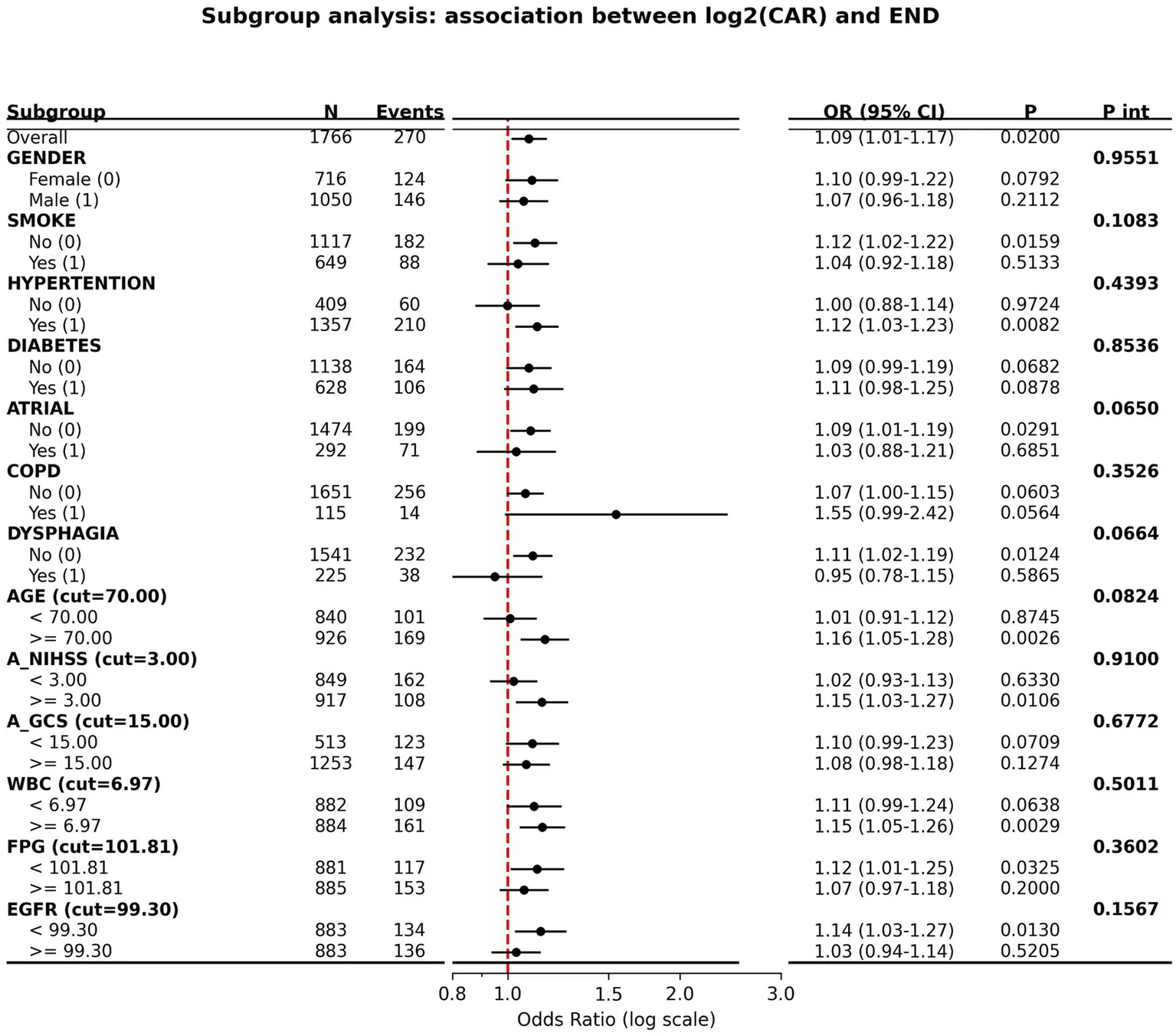
Subgroup analysis of the association between log₂-CAR and END. Odds ratios and 95% confidence intervals for the association between log_2_-CAR and END across prespecified clinical strata. Point estimates were generally positive, but several stratum-specific confidence intervals included 1.0. No interaction test was significant (all *p*-values for interaction >0.05), indicating no evidence of effect modification. Subgroup estimates should not be interpreted as statistically significant within every stratum. CAR, C-reactive protein-to-albumin ratio; END, early neurological deterioration; OR, odds ratio; CI, confidence interval; COPD, chronic obstructive pulmonary disease; A_NIHSS, baseline National Institutes of Health Stroke Scale; A_GCS, baseline Glasgow Coma Scale score; WBC, white blood cell count; FPG, fasting plasma glucose; eGFR, estimated glomerular filtration rate.

**Table 4:** Variance inflation factors (VIFs) for covariates included in the multivariable logistic regression model.

Variable	Description	VIF	Interpretation
log_2_-CAR	Log₂-transformed CAR (primary exposure)	1.4	Low
NIHSS	NIH Stroke Scale score	2.7	Low
AF	Atrial fibrillation	1.2	Low
Hypertension	Hypertension	1.1	Low
FPG	Fasting plasma glucose (mg/dL)	1.5	Low
Age	Age (years)	1.3	Low
WBC	White blood cell count (*10⁹/L)	1.3	Low
Gender	Sex (male = 1)	1.7	Low
Smoke	Current smoking status	1.7	Low
EGFR	Estimated glomerular filtration rate (ml/min/1.73 m²)	1.2	Low
COPD	Chronic obstructive pulmonary disease	1.1	Low
Diabetes	Diabetes mellitus	1.4	Low
GCS	Glasgow Coma Scale score	2.5	Low
Dysphagia	Dysphagia	1.6	Low

The variance inflation factor (VIF) values were calculated for all covariates included in Model 3 (the fully adjusted model from Table 3). Interpretation criteria: VIF < 5 indicates low multicollinearity (acceptable); VIF 5–10 indicates moderate multicollinearity (caution); VIF ≥ 10 indicates high multicollinearity (problematic). All VIF values in the current model are below 3.0, indicating no significant multicollinearity. The mean VIF is 1.54 (range: 1.05–2.69), confirming that the regression coefficients in the multivariable model are stable and reliable. VIF, variance inflation factor; CAR, C-reactive protein to albumin ratio; NIHSS, National Institutes of Health Stroke Scale; GCS, Glasgow Coma Scale; WBC, white blood cell count; FPG, fasting plasma glucose; eGFR, estimated glomerular filtration rate; COPD, chronic obstructive pulmonary disease; AF, atrial fibrillation; Gender, sex (male = 1, female = 0); Smoke, current smoking status; Diabetes, diabetes mellitus; Dysphagia, dysphagia.

### 3.5 Predictive performance of CAR

ROC analysis showed modest discrimination for CAR (AUC = 0.627; Figure 3). CAR outperformed A2DS2 (AUC = 0.520; *p* < 0.001) but did not differ significantly from CRP (AUC = 0.624; *p* = 0.111), neutrophil count (AUC = 0.616; *p* = 0.604), or WBC (AUC = 0.606; *p* = 0.344). The CAR-AUC was numerically 0.003 higher than the CRP-AUC, but this difference was not statistically significant (Table 5).

**Figure 3:**
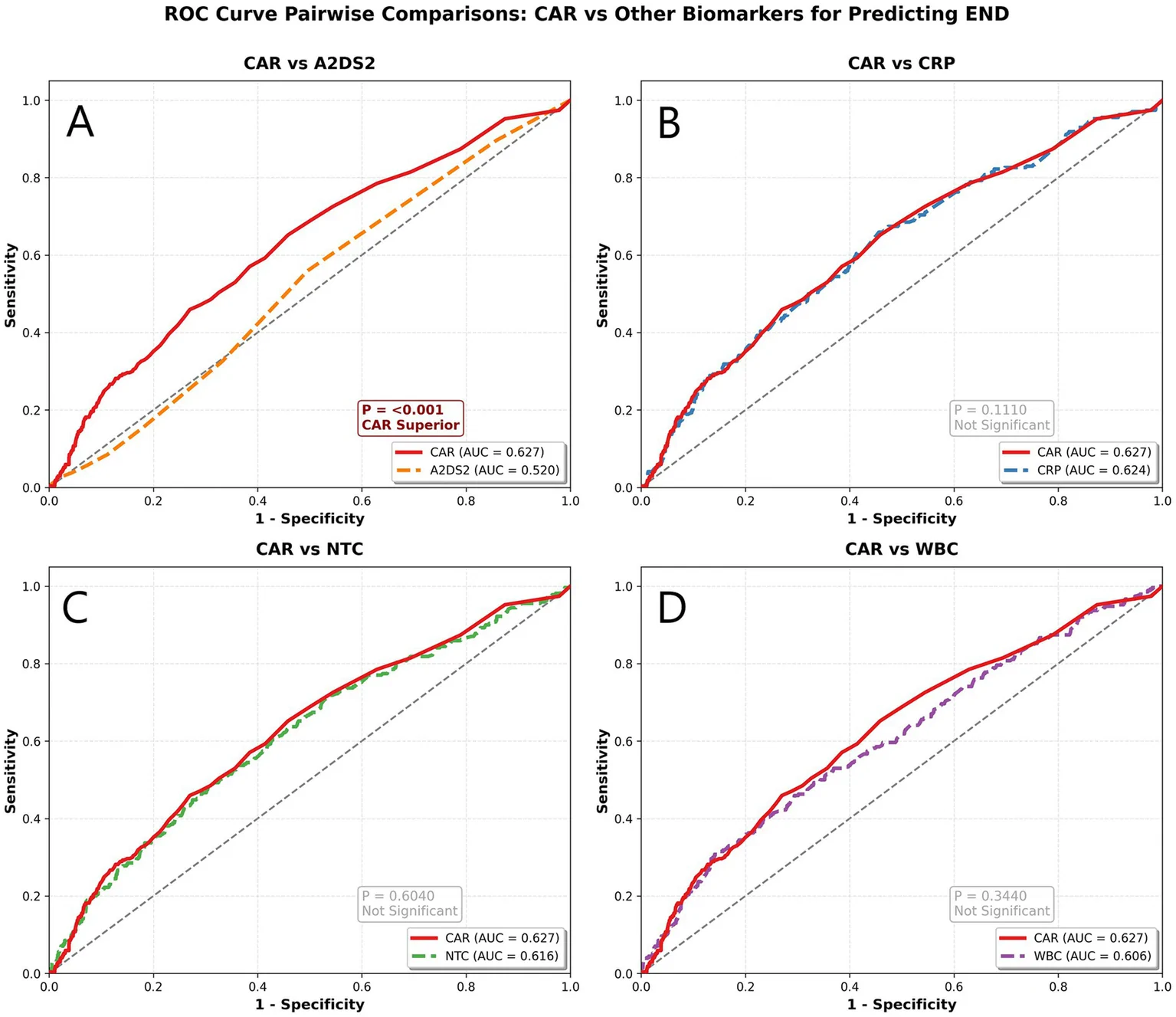
ROC curve pairwise comparisons: CAR vs. other biomarkers for predicting END. Pairwise ROC comparisons for END prediction. **(A)** CAR (AUC = 0.627) vs. A2DS2 (AUC = 0.520), *p* < 0.001. **(B)** CAR vs. CRP (AUC = 0.624), *p* = 0.1110. **(C)** CAR vs. neutrophil count (AUC = 0.616), *p* = 0.6040. **(D)** CAR vs. WBC (AUC = 0.606), *p* = 0.3440. CAR outperformed A2DS2 but was not statistically distinguishable from the three laboratory markers. ROC, receiver operating characteristic; AUC, area under the curve; CAR, C-reactive protein-to-albumin ratio; END, early neurological deterioration; A2DS2, Age–Atrial fibrillation–Dysphagia–Sex–Stroke severity score; CRP, C-reactive protein; NTC, neutrophil count; WBC, white blood cell count.

**Table 5:** Results of statistical tests for pairwise comparisons of ROC curves.

Comparison (A vs B)	AUC (A)	AUC (B)	*p*-value* (DeLong test)	Conclusion
CAR vs A2DS2	0.627	0.52	<0.001	Superior
CAR vs CRP	0.627	0.624	0.1110	Not significant
CAR vs WBC	0.627	0.606	0.3440	Not significant
CAR vs NTC	0.627	0.616	0.6040	Not significant

*p values were derived from DeLong’s test for comparing two receiver operating characteristic (ROC) curves using bootstrap resampling (2,000 iterations). Pairwise comparisons were performed to evaluate whether CAR showed superior diagnostic performance compared to other biomarkers for predicting early neurological deterioration (END). CAR demonstrated significantly better performance than A2DS2, but did not differ significantly from CRP (*p* = 0.111), WBC (*p* = 0.344), or NTC (*p* = 0.604). AUC, area under the receiver operating characteristic curve; ROC, receiver operating characteristic; CAR, C-reactive protein to albumin ratio; A2DS2, Age-Atrial fibrillation-Dysphagia-Sex-Stroke severity score; CRP, C-reactive protein; WBC, white blood cell count; NTC, neutrophil count; END, early neurological deterioration.

Adding CAR improved the AUC of A2DS2 from 0.520 to 0.591 (ΔAUC = 0.071; *p* = 0.0228), WBC from 0.606 to 0.614 (ΔAUC = 0.008; *p* = 0.0223), and neutrophil count from 0.616 to 0.622 (ΔAUC = 0.006; *p* = 0.0169) (Figure 4). In contrast, adding CAR to CRP increased the AUC only from 0.624 to 0.626 (ΔAUC = 0.002), and this comparison was not statistically significant (*p* = 0.0564). Thus, the clearest incremental discrimination was observed when CAR was added to A2DS2, rather than to CRP (Table 6).

**Figure 4:**
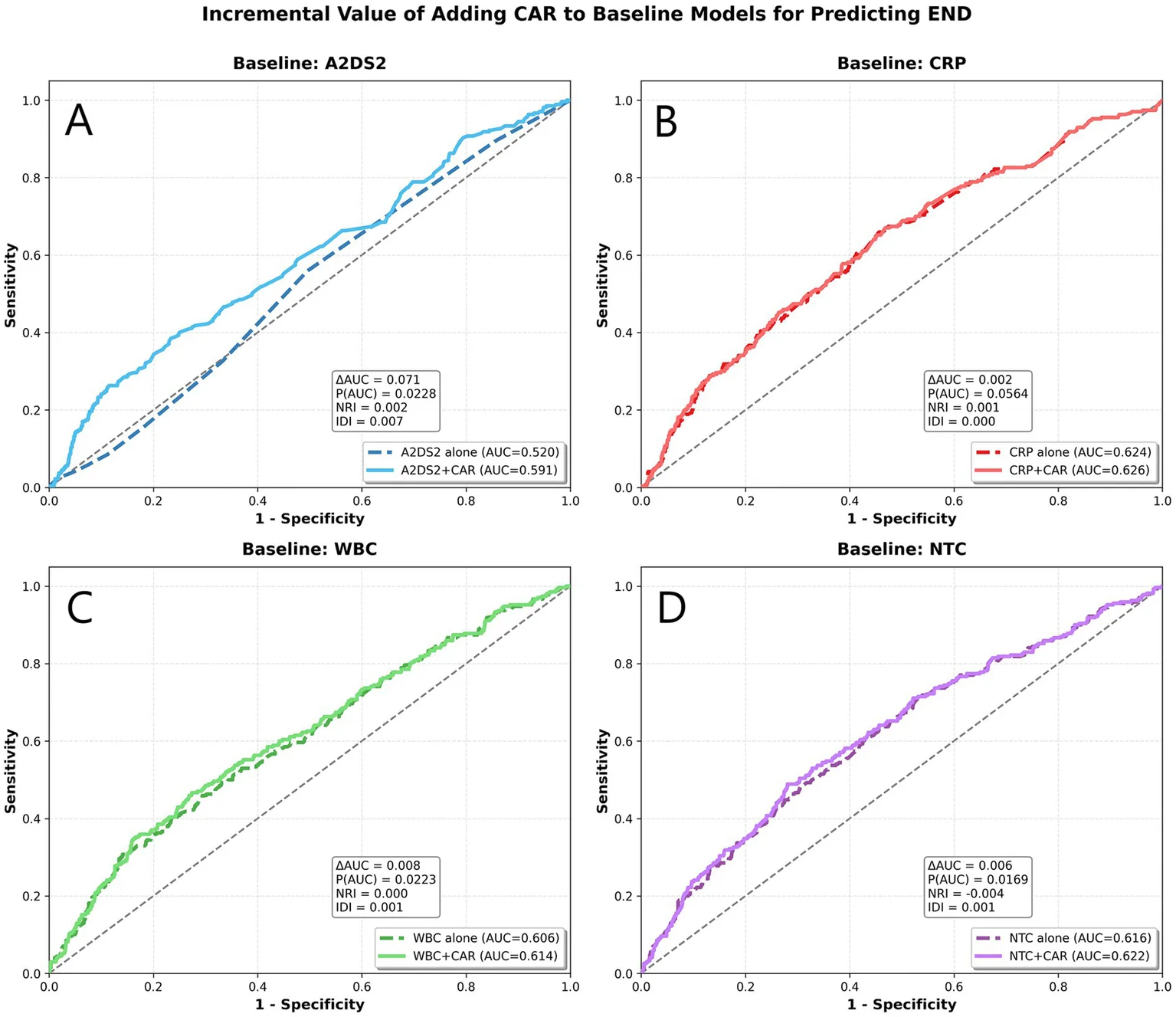
Incremental value of adding CAR to baseline models for predicting END. Incremental ROC comparisons after adding CAR to baseline models. **(A)** A2DS2: AUC 0.520 to 0.591 (ΔAUC = 0.071; *p* = 0.0228). **(B)** CRP: AUC 0.624 to 0.626 (ΔAUC = 0.002; *p* = 0.0564). **(C)** WBC: AUC 0.606 to 0.614 (ΔAUC = 0.008; *p* = 0.0223). **(D)** Neutrophil count: AUC 0.616 to 0.622 (ΔAUC = 0.006; *p* = 0.0169). The largest improvement was relative to A2DS2; improvement beyond CRP was small and not statistically significant. ROC, receiver operating characteristic; AUC, area under the curve; △AUC, incremental change in AUC; CAR, C-reactive protein-to-albumin ratio; END, early neurological deterioration; A2DS2, Age-Atrial fibrillation-Dysphagia-Sex-Stroke severity score; CRP, C-reactive protein; WBC, white blood cell count; NTC, neutrophil count; NRI, net reclassification improvement; IDI, integrated discrimination improvement.

**Table 6:** Incremental value of adding CAR to baseline models for predicting END.

Predictor(s) in baseline model	AUC (baseline)	AUC (baseline + CAR)	*p*-value (AUC)	NRI (95% CI)*	IDI (95% CI)*
A2DS2	0.52	0.591	0.0228	0.002 (−0.0034 to 0.0108)	0.007 (0.0022 to 0.0118)
CRP	0.624	0.626	0.0564	0.001 (0.0000 to 0.0020)	0.000 (−0.0005 to 0.0005)
WBC	0.606	0.614	0.0223	0.000 (−0.0020 to 0.0020)	0.001 (−0.0008 to 0.0031)
NTC	0.616	0.622	0.0169	−0.004 (−0.0142 to 0.0000)	0.001 (−0.0009 to 0.0028)

*p-values for AUC comparisons were calculated using DeLong’s test. NRI (net reclassification improvement) and IDI (integrated discrimination improvement) with 95% confidence intervals (CIs) were calculated to assess the incremental predictive value of adding CAR to each baseline model for predicting early neurological deterioration (END). AUC, NRI, and IDI were estimated using bootstrap resampling (2,000 iterations). AUC, area under the receiver operating characteristic curve; CAR, C-reactive protein to albumin ratio; A2DS2, Age-Atrial fibrillation-Dysphagia-Sex-Stroke severity score; CRP, C-reactive protein; WBC, white blood cell count; NTC, neutrophil count; END, early neurological deterioration; NRI, net reclassification improvement; IDI, integrated discrimination improvement; CI, confidence interval.

### 3.6 Exploratory assessment of non-linearity and a possible threshold

Exploratory generalized additive and two-piecewise analyses suggested a possible change in slope near log₂-CAR = −5.64, corresponding to a raw CAR of approximately 0.020 (Figure 5; Table 7). Below this candidate point, the OR was 2.35 (95% CI, 0.99–5.58; *p* = 0.0524); at or above it, the OR was 1.15 (95% CI, 1.05–1.26; *p* = 0.0025). However, the difference between the segment-specific slopes was not significant (*p* = 0.1131), and the threshold model did not significantly improve model fit over the linear model (likelihood-ratio *p* = 0.0874). These results do not establish a non-linear or threshold-dependent association and should be viewed as hypothesis-generating.

**Figure 5:**
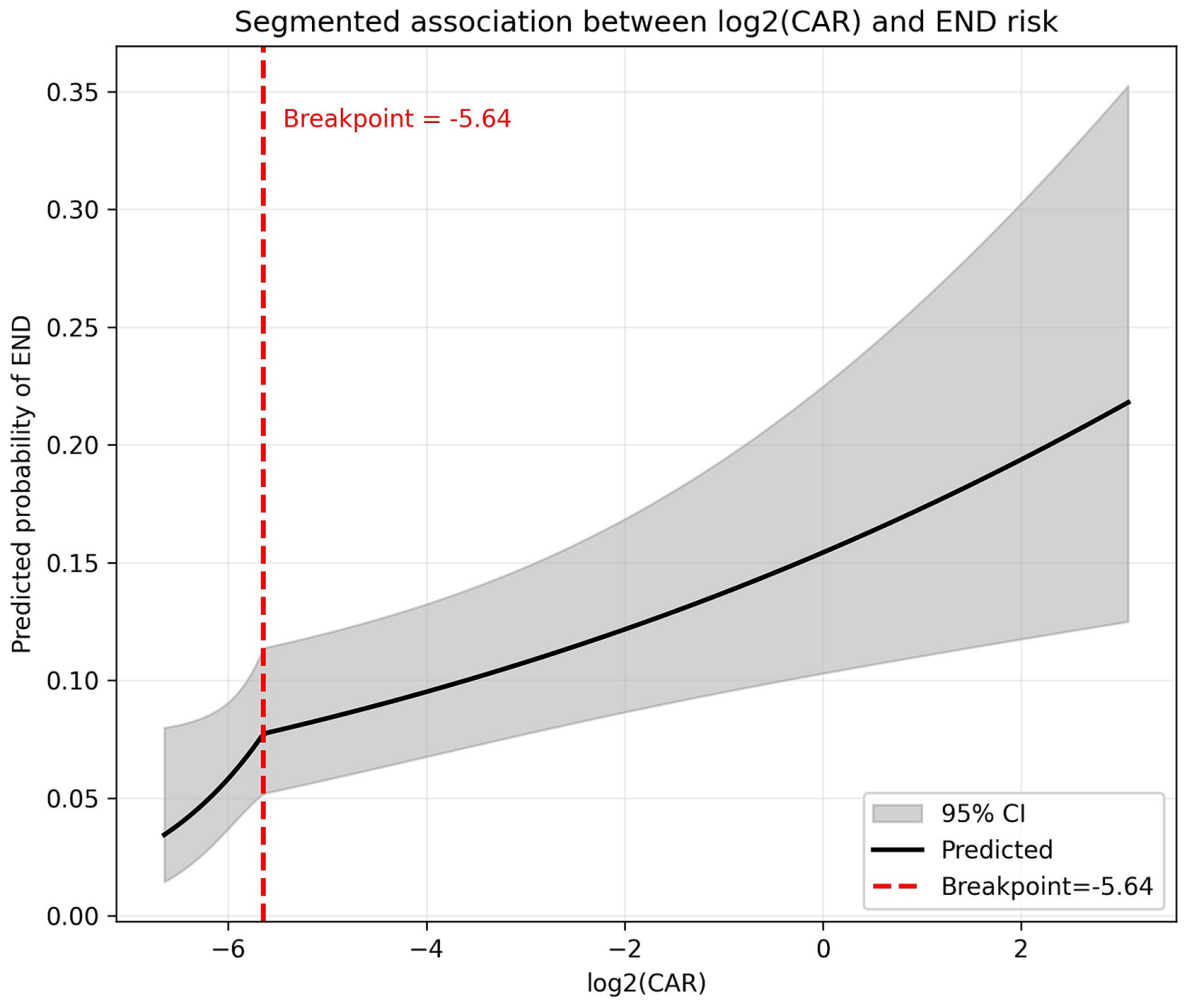
Exploratory segmented association between log₂-CAR and END risk. The solid line shows the model-estimated probability of END, and the shaded band shows the 95% *CI*. The dashed line marks the candidate inflection point at log_2_-CAR = −5.64, corresponding to a raw CAR of approximately 0.0200, from an exploratory two-piecewise logistic regression analysis. Below the candidate point, the adjusted *OR* was 2.35 (95% *CI*, 0.99–5.58; *p* = 0.0524); at or above it, the adjusted *OR* was 1.15 (95% *CI*, 1.05–1.26, *p* = 0.0025). The segment slope difference was not significant (*p* = 0.1131), and the two-piecewise model did not significantly improve fit over the linear model (likelihood-ratio *p* = 0.0874). Therefore, the figure suggests a possible pattern but does not establish non-linearity or a clinically actionable threshold. Models were adjusted for the prespecified demographic, vascular, laboratory, and stroke-severity covariates. The candidate value should be independently validated before clinical use. CAR, C-reactive protein-to-albumin ratio; END, early neurological deterioration; GAM, generalized additive model; *OR*, odds ratio; *CI*, confidence interval.

**Table 7:** Exploratory threshold and linear models for the association between log₂-CAR and END.

END Outcome	Adjusted OR	95% CI	*p*-value
Linear association (log₂-CAR)	1.18	1.09–1.28	**<0.001**
Threshold model (Model II)			
Inflection point	−5.64	CAR ≈ 0.0200	
log₂-CAR < −5.64	2.35	0.99–5.58	0.0524
log₂-CAR ≥ −5.64	1.15	1.05–1.26	**0.0025**
Difference (Segment 2-1)	0.49	0.20–1.19	0.1131
Log likelihood ratio test			0.0874

Multivariable logistic regression models evaluated the linear association and an exploratory two-piecewise model with a candidate inflection point. Estimates were adjusted for age, sex, smoking, hypertension, diabetes mellitus, atrial fibrillation, COPD, dysphagia, admission NIHSS, admission GCS, WBC, FPG, and eGFR. The two-piecewise model did not significantly improve fit over the linear model (likelihood-ratio *p* = 0.0874), and the segment-slope difference was also not significant (*p* = 0.1131). Accordingly, the candidate point at log₂-CAR = −5.64 (raw CAR ≈ 0.0200) should be interpreted as exploratory rather than as a validated threshold. Bold values indicate *p* < 0.05 for individual coefficients. CAR, C-reactive protein to albumin ratio; END, early neurological deterioration; OR, odds ratio; CI, confidence interval.

## 4 Discussion

In this single-center retrospective cohort of 1,766 patients with AIS, higher CAR was independently associated with END. CAR showed modest discrimination and performed better than the A2DS2 score, but it did not significantly outperform CRP. Exploratory modeling suggested a possible change in slope near log₂-CAR = −5.64 (raw CAR of approximately 0.020); however, formal model comparison did not establish non-linearity. The principal finding is therefore an adjusted association between CAR and END, with potential incremental value relative to A2DS2 rather than proven superiority over CRP.

### 4.1 Mechanistic insights

The observed adjusted association between CAR and END may reflect several interconnected mechanisms. Elevated CRP marks systemic inflammation, which can contribute to blood–brain barrier disruption, cerebral edema, and leukocyte infiltration after ischemic stroke (12, 13). Lower albumin may reflect nutritional status, chronic disease burden, or reduced antioxidant and anti-inflammatory capacity. Albumin also contributes to oncotic pressure and platelet regulation (35, 36). These mechanisms are biologically plausible but cannot be established by the present observational analysis.

CAR combines CRP and albumin in a single ratio and may capture information related to inflammation and albumin status. In this cohort, however, conceptual breadth did not translate into a statistically significant discrimination advantage over CRP alone: CAR and CRP had AUCs of 0.627 and 0.624, respectively (*p* = 0.111), and adding CAR to CRP produced ΔAUC = 0.002 (*p* = 0.0564). CAR’s clearest incremental value was observed relative to the A2DS2 clinical score. Accordingly, our data support CAR as an associated composite marker but not as a biomarker proven to outperform CRP.

### 4.2 Comparison with previous literature

Previous studies have examined CRP and albumin as prognostic markers after stroke (37, 38), whereas fewer have evaluated CAR specifically for END. Our results extend this literature by showing an adjusted association with END. CAR was comparable, rather than consistently superior, to CRP, neutrophil count, and WBC in pairwise AUC comparisons. Its practical advantages are that it is easily calculated from routinely available tests and may complement a clinical score such as A2DS2.

The exploratory smooth and segmented models suggested possible curvature, but the likelihood-ratio test comparing the threshold and linear models was not significant (*p* = 0.0874). The candidate point at log₂-CAR = −5.64 (raw CAR of approximately 0.020) should therefore not be interpreted as a validated biological or clinical threshold.

### 4.3 Clinical implications

The results support further evaluation of CAR as an inexpensive adjunct to clinical assessment. Its clearest improvement was observed when added to A2DS2, whereas no significant improvement was observed when added to CRP alone. Because overall discrimination remained modest and the proposed raw-CAR value of approximately 0.020 was exploratory, neither CAR nor this candidate cut point should currently be used as a stand-alone decision threshold for monitoring or treatment.

CAR is derived from routine CRP and albumin measurements and therefore requires no additional assay. Nevertheless, the observational design does not show that interventions aimed at lowering inflammation or increasing albumin would reduce END; that hypothesis requires prospective interventional testing.

### 4.4 Limitations

Several limitations should be acknowledged. First, the retrospective, single-center design limits generalizability and precludes causal inference. Selection bias may exist despite the rigorous inclusion and exclusion criteria. Second, CRP and albumin were measured only once within 48 h of admission; serial measurements might better capture dynamic changes in inflammatory and nutritional status. Third, while we adjusted for numerous potential confounders, residual confounding by unmeasured variables cannot be excluded.

Fourth, END was defined by NIHSS change and may not capture every dimension of neurological deterioration. Fifth, CAR did not significantly outperform CRP alone, and adding CAR to CRP produced only a small, non-significant AUC increase; thus, the main predictive benefit was relative to A2DS2, which limits any framing of CAR as a biomarker that is superior to CRP. Sixth, the likelihood-ratio test did not confirm the proposed threshold model, so the candidate raw-CAR value of approximately 0.020 is exploratory. Seventh, the institutional registry overlaps with previously reported stroke-associated pneumonia cohorts (31, 32), and exact patient-level overlap has not been quantified. Finally, long-term outcomes were not assessed, and external validation is needed.

### 4.5 Future directions

Prospective multicenter studies should validate the adjusted CAR–END association, directly compare CAR with CRP, and assess calibration and clinical utility in external cohorts. Any proposed non-linear relationship or candidate value near raw CAR of approximately 0.020 should be tested prospectively before use as a clinical cut point. Serial CAR measurements and mechanistic studies may also clarify the temporal and biological relationships.

## 5 Conclusion

Higher CAR was independently associated with END in this single-center AIS cohort. CAR showed modest discrimination, and its clearest incremental value was relative to A2DS2; superiority over CRP alone was not demonstrated. Exploratory analyses suggested a possible change in slope near log₂-CAR = −5.64 (raw CAR of approximately 0.020), but formal model comparison did not establish a threshold effect. External validation is required before CAR, or this candidate value, can be used to guide clinical decisions.

## Data Availability

The original contributions presented in the study are included in the article/supplementary material. The raw datasets generated and analyzed during the current study are not publicly available due to ethical restrictions and participant confidentiality, but are available from the corresponding author upon reasonable request, subject to approval from the Institutional Ethics Committee.
